# Global contribution of statistical control charts to epidemiology monitoring: A 23-year analysis with optimized EWMA real-life application on COVID-19

**DOI:** 10.1097/MD.0000000000038766

**Published:** 2024-07-05

**Authors:** Muhammad Waqas, Song Hua Xu, Muhammad Usman Aslam, Sajid Hussain, Khurram Shahzad, Gilbert Masengo

**Affiliations:** a Department of Statistics, School of Mathematics and Statistics, Xian Jiaotong University, Xian, China; b Department of Statistics, University of WAH, Pakistan; c Department of Health Management & Institute of Medical Artificial Intelligence, The Second Affiliated Hospital, Xi’an Jiaotong University, Xian, China; d Yale University, New Haven, CT; e SysReforms International, Department Health Monitoring, Pakistan; f Monitoring and Evaluation Department, Chemonics International Inc., Islamabad, Pakistan; g Department of Mechanical Engineering, Rwanda Polytechnic/Integrated Polytechnic Regional College Karongi, Kigali, Rwanda.

**Keywords:** applications, control charts, COVID-19, epidemiology, global, healthcare, monitoring

## Abstract

Control charts help epidemiologists and healthcare professionals monitor disease incidence and prevalence in real time, preventing outbreaks and health emergencies. However, there remains a notable gap in the comprehensive exploration and application of these techniques, particularly in the context of monitoring and managing disease outbreaks. This study analyses and categorizes worldwide control chart applications from 2000 to 2023 in outbreak monitoring in over 20 countries, focusing on corona-virus (COVID-19), and chooses optimal control charts for monitoring US COVID-19 death waves from February 2020 to December 2023. The systematic literature review analyzes available 35 articles, categorizing data by year, variable, country, study type, and chart design. A selected optimal chart is applied to monitor COVID-19 death patterns and waves in the USA. Control chart adoption in epidemiology monitoring increased during the COVID-19 pandemic, with annual patterns showing a rise in 2021 to 2023 (18%, 36%, 41%). Important variables from 2000 to 2019 include influenza counts, *Salmonella* cases, and infection rates, while COVID-19 studies focus more on cases, infection rates, symptoms, and deaths. Among 22 countries, the USA (29%) is the top applier of control charts. The monitoring of USA COVID-19 deaths reveals 8 waves with varying severity $W_{5}\left(1899\right)$ > $W_{3}\left(1881\right)$ > $W_{4}\left(1393\right)$ > $W_{1}\left(1036\right)$ > $W_{2}\left(853\right)$ > $W_{6}\left(478\right)$ > $W_{7}\left(140\right)$ > $W_{8}$. The $W_{8}$ associated with the JN.1 variant, highlights ongoing challenges. This study emphasizes the significance of control charts in outbreak monitoring for early disease diagnosis and intervention. Control charts help healthcare workers manage epidemics using data-driven methods, improving public health. COVID-19 mortality analysis emphasizes their importance, encouraging worldwide use.

## 1. Introduction

Epidemiology underpins public health and healthcare administration. This field focuses on identifying disease burdens, determining priorities, assessing cause-and-effect correlations, developing solutions, monitoring, and evaluating healthcare.^[1]^ Devastating pandemics have occurred throughout history such as the Plague, Spanish Flu, HIV, and Ebola causing deaths, political government collapse, and financial and psychosocial hardships. We have seen immense anguish, mortality, and instability in everyday life for more than 4 years during the corona-virus (COVID-19) pandemic.^[2]^ Human populations could be severely burdened by emerging viruses in terms of death, morbidity, and economic costs. The examination of case data collected during an outbreak has historically been used to monitor the spread of infectious illnesses and aid in their containment.^[3]^

Control charts aid in the early detection of pandemics by tracking the number of cases and deaths of a certain illness over time; any significant increase in the number of cases and deaths might indicate an outbreak. Control charts assist in monitoring process reliability and efficacy over time and discovering notable changes or trends that may indicate the need for intervention. They could serve as indicators of the occurrence of infectious illnesses.^[4–9]^ Moreover, disease monitoring allowed researchers to ascertain seasonal or cyclical patterns in the incidence of the condition as well as if it was growing or decreasing. The effectiveness of an intervention in reducing the number of incidents before and after implementation may be ascertained by intervention evaluation. The quality of medical facilities may be monitored and enhanced using quality improvement techniques.^[10–13]^

Worldwide, respiratory syncytial virus is a major cause of illness and death in young children.^[14]^ Due to prolonged suboptimal viral exposure, immunologically vulnerable older children had more symptomatic respiratory syncytial virus infections and hospitalizations in 2021 to 22 and 2022 to 23 in British Columbia.^[15]^ Acute COVID-19 was the main trait linked with high severity and death in Pakistani children.^[16]^ Numerous academics have examined data on COVID-19 hospitalizations and deaths throughout different waves; such as COVID-19 deaths in the Western Pacific,^[17]^ COVID-19 among the top 5 causes of deaths in Australia,^[18]^ and COVID-19 cases and deaths in the USA.^[19–22]^ Furthermore, in the context of COVID-19 death monitoring by using control charts following studies proposed different charts and identified death patterns.^[23–27]^

Medical information determines control chart classification. There may be continuous, count, or attribute-based data. For instance, attribute control charts provide data with limited values, such as disease presence or absence. Many settings may employ the P, NP, and U control charts. Event-counting data, such as the number of unfavorable occurrences, may be utilized with count data control charts like the C and Histogram charts. Continuous data control charts display statistics like blood pressure, heart rate, and body temperature within a set range. Additionally, there are 2 scenarios: individual moving range control charts and MR X, R, and S. Future, plans include creating control charts for time series data, such as monthly patient satisfaction ratings or daily hospital admissions. Additionally, run charts and time-weighted control charts also exist.^[28–33]^ Rising trend in machine learning techniques in healthcare quality monitoring is observed.^[34–37]^ Choosing the right control chart for the data may prevent inaccurate inferences regarding a healthcare process’s stability.

## 2. Research gap and study significance

How did the world’s most prosperous country, ranked first in its ability to respond to pandemics according to the Global Health Security Index, see more than 1·2 million people die from COVID-19 and have one of the highest rates of death per capita in the world. The importance of evidence-based monitoring and decision-making is emphasized by the study.^[38]^ This study addresses a notable gap in understanding the evolving landscape of control charts in epidemiology monitoring. While there are systematic reviews on the topic of control chart applications in healthcare in general, they frequently do not provide a comprehensive view of control chart applications in epidemiology. Furthermore, many applications contributed to monitoring COVID-19 variables during 2019 to 2023, but an analysis of how those studies contributed overall is also lacking. Furthermore, a debate and the need for a comprehensive analysis of all-time applications were felt to be fulfilled for the selection and application of optimum control charts^[23]^ and their appropriateness in monitoring epidemiological phenomena. This article aims to contribute to the body of knowledge by thoroughly examining the applications of control charts in epidemiological surveillance and global trends in the past 2 decades.

Monitoring gaps impose a burden on the health sector. During an extensive review, we discovered that while the USA is the largest user of control charts in all of healthcare and epidemiology, Shewhart charts are used 90% of the time. Other types of charts that suit better than Shewhart charts should be introduced for monitoring. According to data sources (United States COVID-19 Coronavirus Statistics from Worldometer [worldometers.info]) and (https://covid.cdc.gov/covid-data-tracker/#datatracker-home) the number of cases and deaths are still significant, and there is a significant positive correlation exists between number of cases and deaths in the USA situation.^[23]^ Since, December 9, 2023, the JN.1 variant has surged, the number of cases rising again. The goal of this study is to extend the use of optimal control charts in epidemiology to monitor variations in COVID-19 deaths to better understand recent pandemic mortality patterns. The application of an appropriate statistical process monitoring technique for distinct deaths in the USA in 8 phases, including pregrowth, and postgrowth, is an essential aspect of the current study.

## 3. Methodology

### 3.1. Method 1

#### 3.1.1. Systematic review

Employing the guidelines and methods for the systematic evaluation of QI interventions^[28]^ and Johan, Jonas, Jakob, Jesper, Cheryl, Karin Pukk, and Mats,^[39]^ an extensive search was conducted to uncover materials published between 2000 and 2023 about establishing control charts in the epidemiology. SCOPUS, PubMed, Web of Science, and Google Scholar, databases are used to identify research that examines control charts in monitoring any outbreaks. The search terms included “SPC,” “Control chart,” “Epidemiology,” “COVID-19,” “Application,” and “Outbreaks” etc. Because the emphasis was on locating studies published in academic and professional journals, master’s and doctorate dissertations were eliminated.

#### 3.1.2. Selection of studies and data collection

The title and abstract were reviewed to determine if the paper matched the inclusion criteria. A control chart application in the epidemiology department is the inclusion standard. The required articles were read carefully under the instruction of.^[40]^ Articles that did not match the requirements were discarded. Title, authors, year of publication, location, inclusion criteria, research aims, results, output variables, journal, unit of analysis, study setting and level, and statistical process control (SPC) chart data period were entered in an Excel sheet. Visual statistics in graphs showed country and publication year. SPC in any epidemic research objectives, outcomes, limits, and benefits were qualitatively analyzed in the review.

We approached 2 SPC experts, 1 of whom is the most prominent in the healthcare monitoring engaged with Chemonics Inc (Islamabad, Pakistan). The other is a specialist in SPC application outside of healthcare, to review an earlier draft of this manuscript to improve our review through investigator triangulation.^[41]^ Their feedback improved our data synthesis and reduced findings.

In this evaluation, research papers must apply control charts to epidemiology departments. After reviewing abstracts, 90 of 139 articles were deleted as irrelevant, review papers, tutorials, or used in psychiatric or other health departments. From the remaining 49 research papers, after carefully reading in the full-text form a certain number of 35 studies (14 other outbreaks, 21 COVID-19 studies) are chosen for comprehensive examination and review, see Figure S1, Supplemental Digital Content, http://links.lww.com/MD/N101.

### 3.2. Method 2

#### 3.2.1. Data and settings

The Epidemic Intelligence from Open Sources initiative, which is led by the World Health Organization, signifies an unparalleled coalition of various public health stakeholders from around the globe. The daily COVID-19 deaths in the USA are obtained from the portal, https://portal.who.int/report/eios-covid19-counts. The data spans the periods of February 2020 to December 2020 to 2023. A thorough examination revealed that an optimal.

Exponentially weighted moving average (EWMA) control chart outperformed both the Shewhart and cumulative sum charts in monitoring deaths in the USA. Following the conclusions given in,^[23]^ rather than reproducing the aforementioned methodology, we chose the EWMA chart to extend COVID-19 death wave monitoring in the USA. This decision was influenced by the EWMA chart’s superior performance when compared to cumulative sum, which has known limits,^[42]^ and Shewhart (X-bar, R and C) charts, which performed poorly for the USA situation. The mathematical structure of the EWMA chart is provided in the Method 2, Supplemental Digital Content, http://links.lww.com/MD/N101. We estimated process parameters using wave-1 data. Simulations were run with a range of L values using these estimates. L is used when the average run length is 370, indicating the expected number of observations before a signal is identified. The targeted degree of sensitivity for detecting process changes was *L* = 2.87 to ensure that the control chart successfully recognizes any substantial deviations from expected behavior. The following tools; such as draw.io, Microsoft Excel 16, Origin 9.0, Minitab 21, and R 4.3.2 Language, were utilized in the analysis, graphic work, and simulations of this study.

## 4. Results and discussion

### 4.1. Stage 1

**Figure 1.**
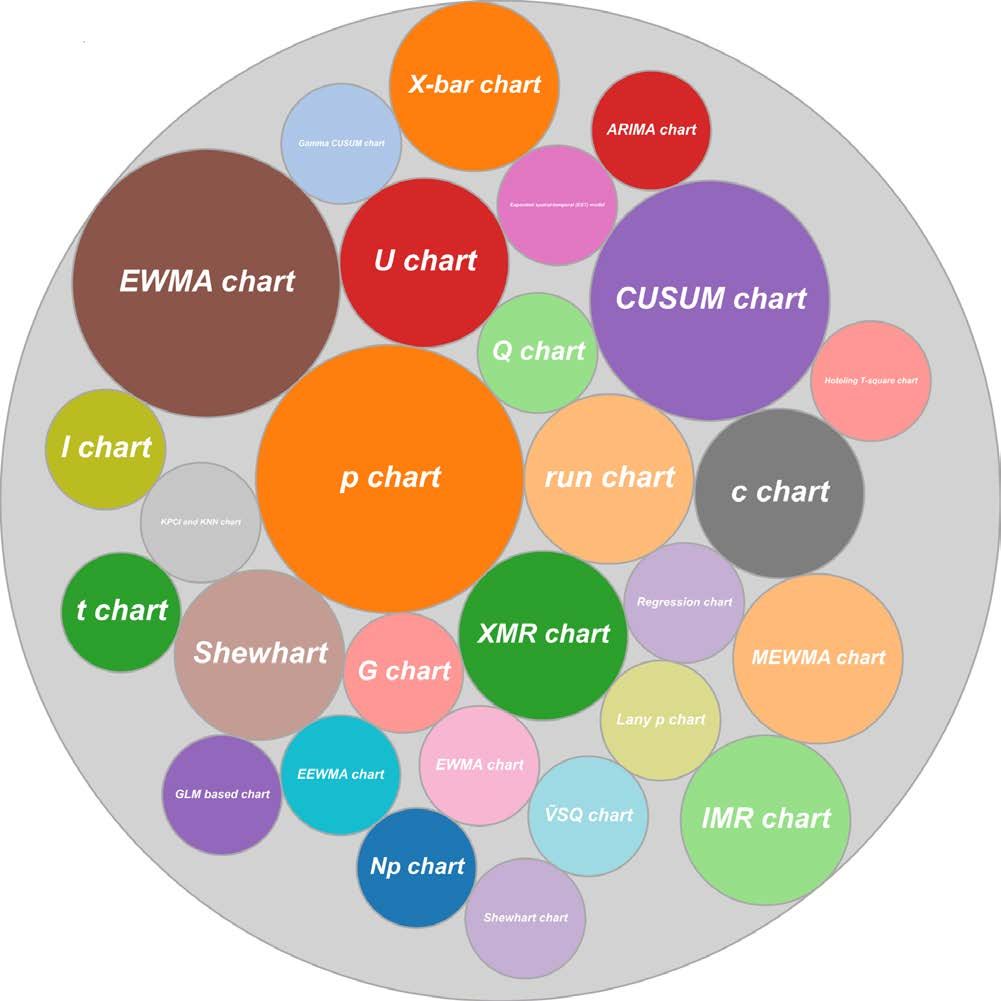
Illustrating the names of each chart which are applied once or more times in any epidemiological monitoring.

**Figure 2.**
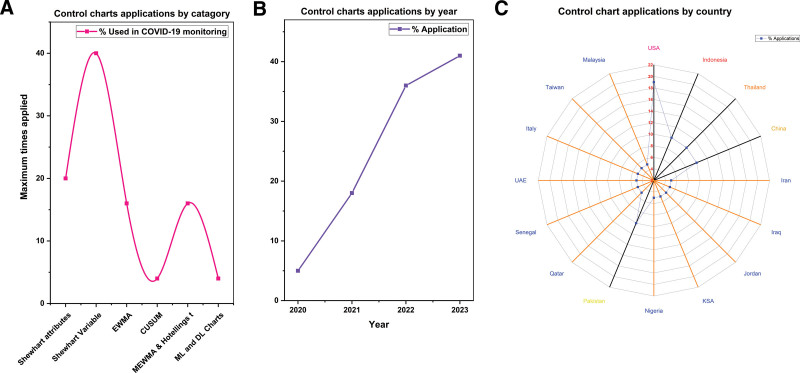
Summarizing the control chart applications in corona-virus (COVID-19). (A) Catagory-wise, (B) year-wise, (C) by country.

**Figure 3.**
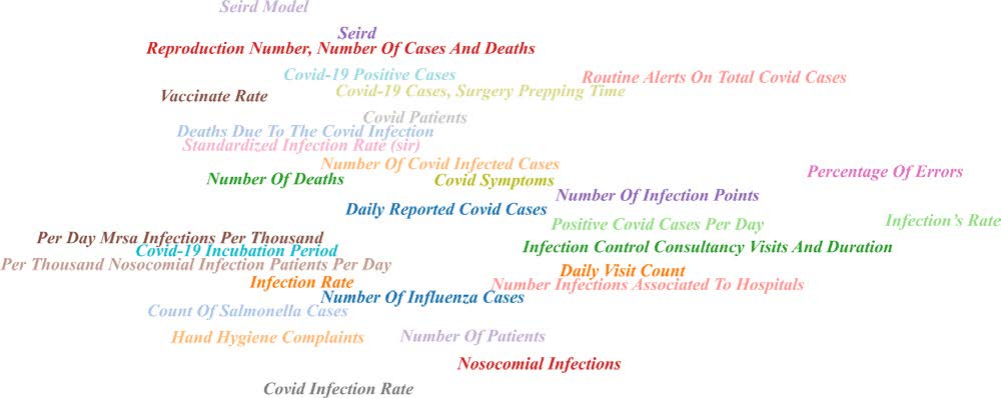
Mostly studied variables in control chart applications.

After analyzing all 35 studies, it was determined that, overall, between 2000 and 2023, the majority of attention was still focused on the recent pandemic, which accounted for 60% of control chart applications between 2020 and 2023, while the other outbreaks received 40% of applications between 2000 and 2019. A detailed summary of each article is presented, see Table 1 and Figures 1–3. Epidemiology control chart applications vary by country, demonstrating worldwide infectious disease surveillance methodologies. The US leads epidemiologically with 29% of control chart applications, followed by Australia at 5%, Brazil, Indonesia, China, and Thailand at 5% each. Control charts are common in the US, indicating their use in epidemiological monitoring. Switzerland, France, the UK, Senegal, Pakistan, Iraq, Jordan, the UAE, Nigeria, KSA, Qatar, Iran, Italy, Malaysia, and Taiwan account for different percentages in Europe, the Middle East, and Asia. The distribution of control chart applications changes for COVID-19 monitoring. The US accounts for 19% of control chart applications. The other countries-specific findings also highlight the necessity for specialized epidemiological monitoring systems to address pandemic problems in varied locales, see Figure 2C.

**Table 1:** A whole summary of control chart applications in different outbreaks between 2000 and 2023.

Outbreak	Study	Chart	Year	Monitored variables	Category	Country
Influenza, *Salmonella*, infection and other outbreaks	Gustafson^[43]^	p, run, and XMR chart	2000	SIR	Retrospective study	USA
	Quesenberry^[44]^	Q chart		Infection’s rate		
	Hanslik et al^[45]^	U chart	2001	Number of patients	Longitudinal study	France
	Arantes et al^[46]^	p chart	2003	Per thousand nosocomial infection patients per day		Brazil
	Grant and Kim^[5]^	XMR chart	2007	Infection control consultancy visits and duration	Retrospective study	USA
	Curran et al^[10]^	p chart	2008	Percentage of errors		UK
	Harbarth et al^[47]^	Run chart		Per day MRSA infections per thousand		Switzerland
	Limaye et al^[48]^	G, U, and CUSUM chart		Number of infections associated with hospitals	Longitudinal study	USA
	Chimka^[6]^	Regression chart	2009	Number of influenza cases		
	Sparks et al^[49]^	CUSUM and EWMA chart	2010	Daily visit count		Australia
	Gomes et al^[50]^	Shewhart, EWMA, CUSUM chart	2011	Nosocomial infections	Retrospective study	Brazil
	Zhou et al^[51]^	EST model	2014	Number of infection points		China
	Wiemken et al^[52]^	p chart	2017	Hand hygiene complaints		USA
	Vanli and Giroux^[53]^	CUSUM chart	2022	Count of *Salmonella* cases	Longitudinal study	
COVID-19	Yuyun Hidayat and Titi^[54]^	t-chart and I-MR chart	2020	COVID-19 positive cases	Retrospective study	Indonesia
	Mbaye et al^[55]^	p chart	2021	Positive COVID cases per day		Senegal
	Mahmood et al^[24]^	EWMA and c chart		Deaths due to the COVID-19 infection		Pakistan
	Inkelas et al^[56]^	I and c chart		Daily reported COVID cases		USA
	Yupaporn and Rapin^[57]^	EWMA chart		Routine alerts on total COVID cases		Thailand
	Mustafa and Jabir^[58]^	KPCI and KNN chart		Number of COVID-infected cases		Iraq
	Arafah^[59]^	Laney p’, EWMA chart	2022	COVID infection rate		Jordan
	Karoon et al^[60]^	EEWMA chart		COVID-19 cases	Longitudinal and retrospective study	Thailand
	Sanmugam and Abdul^[61]^	Shewhart chart		COVID-19 cases	Longitudinal study	Malaysia
	Fernandez et al^[62]^	X-bar chart		COVID-19 cases, surgery prepping time	Retrospective study	USA
	Barone and Chakhunashvili^[63]^	Individual chart	2023	COVID-19 cases	Longitudinal and retrospective study	Italy
	Faisal Shah and Khan^[64]^	ṼSQ chart		COVID-19 incubation period	Retrospective study	China
	Alkhatib et al^[65]^	Np chart		Infection rate	Longitudinal study	UAE
	Adekeye and Adekeye^[26]^	Gamma CUSUM chart		Number of deaths		Nigeria
	Waqas et al^[23]^	Shewhart and EWMA chart		Reproduction number, number of cases and deaths		USA
	Boone et al^[25]^	MEWMA chart		SEIRD		Qatar
	Imro’ah et al^[66]^	I-MR and ARIMA chart		Vaccinate rate		Indonesia
	Alamri et al^[67]^	MEWMA chart		SEIRD model		KSA
	Elhambakhsh et al^[68]^	Hoteling T-square chart		COVID symptoms		Iran
	Cheema et al^[27]^	GLM based chart		COVID patients	Retrospective study	Pakistan
	Wang^[69]^	X-bar chart		COVID-19 cases		Taiwan

AICE = National Database Initiative USA, CDCP = Center for Disease Control and Prevention USA, CUSUM = cumulative sum, EEWMA = extended exponentially weighted moving average, EST = expanded spatial-temporal, EWMA = exponentially weighted moving average, I-MR = individual moving range, KNN = K-nearest neighbor control chart, KPCI = Kernel principal component analysis chart, MEWMA = multivariate EWMA, MRSA = methicillin-resistant *Staphylococcus Aureus*, NNIS = National Nosocomial Infections Surveillance, SIR = standardized infection rate, t-chart = tangent chart.

### 4.2. Overall interesting inferences

Following intriguing patterns shed light on the diverse strategies employed in epidemiological studies, the specificity in COVID-19 monitoring, and the collaborative global effort to tackle the unprecedented challenges presented by infectious diseases.

#### 4.2.1. USA dominance in the standard control chart

A striking observation is the dominance of standard control charts in the USA, with 90% of applications utilizing charts other than EWMA. This suggests a strong reliance on traditional control chart methodologies, pointing toward a preference for well-established and widely accepted techniques in the country’s epidemiological studies. Other side, this also provides a space for the application of other control charts in epidemiolocal phenomena.

#### 4.2.2. Global adoption of control charts for diverse variables

The diverse range of variables studied using control charts reflects a global trend. Countries such as France, Brazil, Switzerland, China, and the UK employ various control charts for monitoring infection-related variables, showcasing the adaptability of control charts across different nations and epidemiological contexts.

#### 4.2.3. Specificity in COVID-19 monitoring techniques

In COVID-19 studies, there is an efficient approach with specific control charts tailored for distinct variables related to the pandemic. This includes the use of charts such as EWMA & c chart for deaths, p chart for positive COVID cases per day, and Laney p and EWMA chart for COVID infection rate. These tailored approaches emphasize the need for precision in monitoring the unique characteristics of the ongoing global health crisis.

#### 4.2.4. Global collaboration in COVID-19 research

The inclusion of multiple countries in COVID-19 studies, such as the USA, UK, China, Italy, Switzerland, Thailand, Iraq, Jordan, Senegal, Pakistan, and more, suggests a collaborative effort in understanding and combatting the pandemic. This international collaboration underscores the collective response to a global health crisis, leveraging diverse methodologies and chart types to gain comprehensive insights into the impact of COVID-19.

#### 4.2.5. Longitudinal studies for COVID-19 impact

The use of longitudinal studies for COVID-19 variables, such as COVID-19 cases, deaths, reproduction numbers, and surgery prepping time, indicates a keen interest in understanding the long-term impact and trends associated with the pandemic. This longitudinal perspective is crucial for formulating effective strategies for managing and mitigating the ongoing challenges posed by COVID-19.

### 4.3. Stage 2

#### 4.3.1. USA COVID-19 deaths monitoring

**Figure 4.**
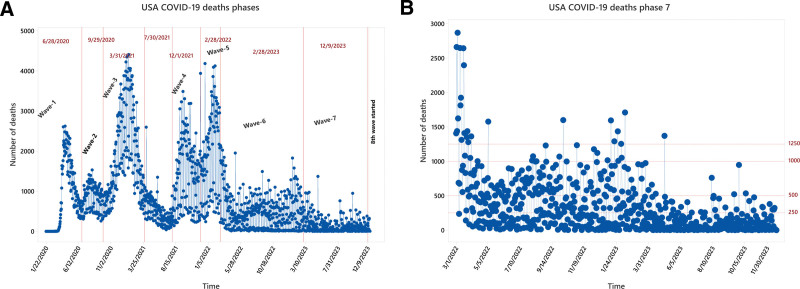
Depicting the wave phase-wise distribution of all-time COVID-19 waves in the USA.

The timeline analysis from 2020 to December 2023, see Figure 4 shows how COVID-19 waves are dynamic and are affected by several variables over time, covering average deaths, number of days, and severe waves, see Table 2. Seasonal fluctuations, adaption to novel varieties, and vaccination campaigns are all important factors in determining how the pandemic develops. Effective public health initiatives and well-informed decision-making depend on an understanding of these temporal trends.

**Table 2:** Wave-wise summary of COVID-19 deaths in the USA.

Time	Mortality waves	Days	Missing	Mean	SE mean	SD	Sum	Minimum	Q1	Median	Maximum
February–June 2020	Initial period deaths	123	0	1036	70.7	784.1	127,430	0.0	379	870	2624
July–September 2020	Second wave	92	0	853	32.6	312.9	78,517	258.0	555	920	1532
October 2020–March 2021	Third wave (vacc. rollout)	182	0	1881	79.7	1075.4	342,395	384.0	992	1600	4408
April–July 2021	Spring/summer	122	0	501	30.4	335.9	61,139	40.0	286	445	2598
August–November 2021	Fourth wave (delta variant)	122	0	1393	78.0	861.2	169,972	140.0	567	1335	3487
December 2021–February 2022	Fifth wave (Omicron variant)	90	0	1899	115	1092	170,889	225	810	1909	4185
March 2022–February 2023	Sixth wave	363	1	478	22.9	436.5	173,650	1.0	82	407	2097
March–November 2023	Seventh wave	285	0	140	11.4	193.1	39,970	0.0	14	65	1370

COVID-19 = corona-virus.

##### 4.3.1.1. Emerging phase

**Figure 5.**
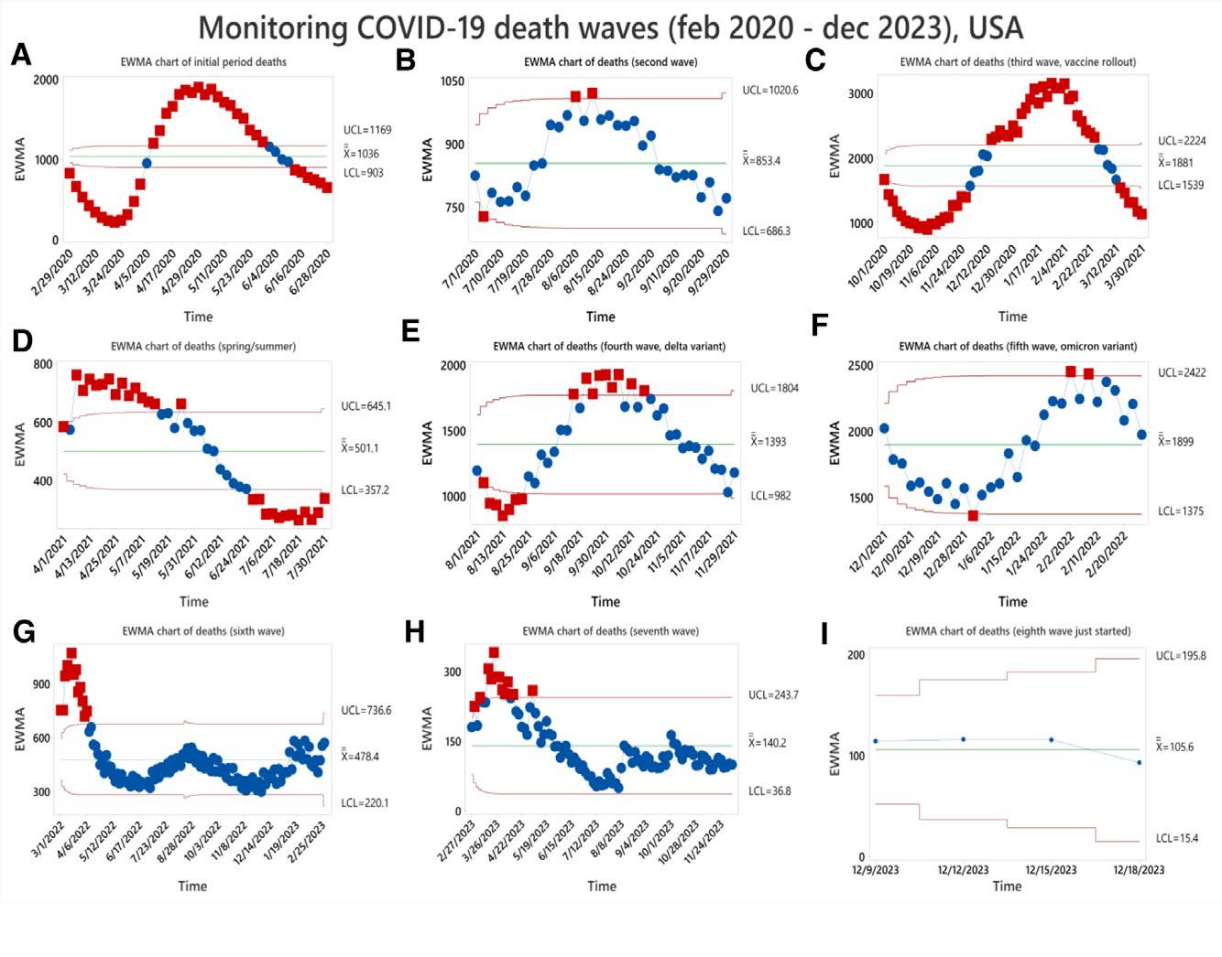
Demonstrating the death waves monitoring by using the EWMA chart. EWMA = exponentially weighted moving average.

The initial stages of the pandemic were symbolized by the first wave, which ran from February to June 2020. Its 123-day lifespan and mean fatalities value of 1036 per day, upper control limit (UCL) = 1169, and peak touch to 2624 deaths in a say demonstrated the early difficulties in comprehending and controlling the new coronavirus, see Figure 5A.

##### 4.3.1.2. Second wave

The mean daily mortality rate dropped to 853.4, UCL = 1020 during the second wave, but 1532 deaths in a single day hit drums, which ran from July to September 2020. This wave, which persisted for 92 days, represented a period of ongoing adjustment and reaction to the changing epidemic, see Figure 5B.

##### 4.3.1.3. Vaccination rollout-third wave

The third wave, which coincided with the vaccine program, lasted 182 days, maximum number of deaths in a single day of 4408, and ran from October 2020 to March 2021. This wave, which had a mean mortality value of 1881.3, UCL = 2224 demonstrated how vaccination campaigns had a positive effect in reducing the virus’s severity, see Figure 5C.

##### 4.3.1.4. Spring/summer wave

The 2021 spring and summer wave had a mean fatalities value of 501.1, UCL = 645, and lasted 122 days. Seasonal changes and ongoing efforts to control the epidemic were evident during this time, see Figure 5D.

##### 4.3.1.5. Fourth wave (delta variant)

The delta variant’s fourth wave had a mean mortality value of 1393.2 per day, UCL = 1804 for 122 days. This wave demonstrated the difficulties brought about by newly developing varieties and the necessity of continuous modification in public health interventions, see Figure 5E.

##### 4.3.1.6. Omicron variant-fifth wave

The Omicron variant-related fifth wave had a significant mean mortality value of 1899 per day, UCL = 2422 and 4185 in a single day, for 90 days. This time frame brought attention to the possible increased severity or transmissibility linked to new variations, see Figure 5F.

##### 4.3.1.7. Sixth wave

With a duration of 363 days, the sixth wave showed a mean death value of 478.4 per day and UCL = 736. This prolonged duration indicated that the pandemic management was still challenging, either because of novel varieties or shifting dynamics, see Figure 5G.

##### 4.3.1.8. The seventh wave

The previous seventh wave, which lasted 285 days, showed a significantly lower mean daily mortality rate of 140.2 and UCL = 243.7. This most recent wave suggests that efforts to contain the virus are still being made, perhaps with better therapies or public health initiatives, see Figure 5H.

##### 4.3.1.9. Eightieth wave; JN.1 variant

Starting from December 9, 2023, a new surge in COVID-19 cases was noticed due to the JN.1 variant. The cases are rising daily which will positively be correlated to an increased number of deaths, see Figure 5I.

Since the beginning of COVID-19, many national and international platforms have begun to collect and manage daily cases, including the number of infected, recovered, and deaths. The data used in this study were nearly complete and efficiently handled. During the analysis, 1 missing value was discovered in the sixth wave data and replaced by the weakly moving average value. The third, fifth, and sixth waves remain the most severe in a total number of deaths more than 686,934. A detailed analysis improves decision-making and reaction strategy formulation for healthcare authorities in pandemic control measures.^[24]^ Rather than only using UCLs, we also applied the EWMA chart lower control limit (LCL). As UCL tells alarming situation, LCL shows a safer situation. Data below the LCL are also evident that the COVID-19 pandemic may be a postgrowth period as mortality has dropped. Positive scores below the LCL imply epidemic decline. The EWMA chart’s LCL can help public health professionals analyze and monitor current conditions. Lockdowns, isolations, vaccines, and other COVID-19 prevention methods are compared to the LCL. By regularly monitoring data points below the LCL, researchers may analyze these indicators and public health strategies to limit infection and reduce deaths.

## 5. Strength and limitations

This study, which spans 23 years and includes 35 studies, provides a robust and extensive analysis of control chart applications in epidemiology. It provides a comprehensive and global perspective by incorporating data from various countries such as the USA, Australia, Brazil, and China, enhancing the generalizability of findings. Notably, the study focuses on COVID-19, in particular, providing detailed insights into its impact on control chart applications.

While this study has strengths, it faces limitations. Variability in data availability and reporting among selected studies may restrict reliance on existing literature. The study’s endpoint in 2023 might overlook emerging trends. The dominance of data from certain countries could impact the broader applicability of findings. Further validation and exploration of the EWMA methodology are needed to assess its effectiveness beyond the USA. These limitations should be considered when interpreting the findings.

## 6. Key takeaways for academia and health experts

First, the study emphasizes the adaptability of traditional control charts to many infectious diseases beyond COVID-19. As epidemiological studies improve precision and efficiency, researchers should incorporate new technologies like machine learning, deep learning, and artificial intelligence (AI) based charts. Despite the growing importance of technology, AI-based charts are scarce, suggesting an area for exploration and development. The study emphasizes longitudinal and retrospective approaches to capture infectious disease trends’ dynamic nature. Health experts say the findings emphasize the need for sophisticated and variable-specific COVID-19 monitoring using tailored control charts for pandemic variables. International epidemiology research requires shared methods and insights to improve pandemic preparedness and response. The study offers actionable insights that can guide future research and suggests that AI may improve epidemiological surveillance.

## 7. Conclusion

In conclusion, this thorough study provides a sophisticated knowledge of epidemiology’s expanding control chart applications, with a focus on the extraordinary COVID-19 pandemic difficulties. According to the study control chart applications increased significantly, peaking during the pandemic, with 60% of studies dedicated to this vital era. The rise in applications in 2021 and 2022 emphasizes the need for control charts to monitor and analyze the global health situation.

Influenza counts, *Salmonella* cases, infection rates, and COVID-19-specific measures remain prominent topics in epidemiological discussions. Control charts’ broad approach to infectious diseases is shown by their applicability to varied epidemiological scenarios and the variety of variables addressed. International collaboration is evident in epidemiological control chart applications, with the US as the leading country. The rise of COVID-19 charts and their varying use across nations highlight the need for pandemic-specific monitoring tools and research collaborations. The study’s second part monitored the 8 COVID-19 death waves in the US. The essential findings from each wave are highlighted for health professionals and policymakers to delve deeply into the outcomes of decisions made over these years. These dynamics demonstrate the effects of vaccination efforts, seasonal variations, and novel mutations. As the virus mutated to JN.1 a sudden spike in cases started which translated into the increased number of deaths. The measures taken before the sixth, fifth, and third waves should be examined and debated to avoid future catastrophes.

The chart is suggested for future applications in other healthcare departments. This study demonstrates to academics, policymakers, and public health practitioners that infectious disease management requires personalized tactics and continual monitoring. The complex dynamics of COVID-19 fatalities waves highlight the necessity for adaptive public health strategies. The suggested tools improve epidemiological monitoring with specialized distributions, adding to global infectious disease control efforts.

## Acknowledgments

The authors thank the Institute of Medical Artificial Intelligence, the Second Affiliated Hospital, and School of Mathematics and Statistics Xi’an Jiaotong University, Xi’an China, and the University of WAH for offering research facilities.

## Author contributions

**Conceptualization:** Muhammad Waqas.

**Data curation:** Muhammad Waqas.

**Formal analysis:** Muhammad Waqas.

**Methodology:** Muhammad Waqas.

**Visualization:** Muhammad Waqas.

**Writing—original draft:** Muhammad Waqas.

**Supervision:** Song Hua Xu.

**Writing—review & editing:** Muhammad Usman Aslam, Sajid Hussain, Gilbert Masengo.

**Validation:** Khurram Shahzad.

## Supplementary Material
