# Supplementary material for: Global contribution of statistical control charts to epidemiology monitoring: A 23-year analysis with optimized EWMA real-life application on COVID-19
Source: Medicine (Baltimore). 2024 Jul 5;103(27):e38766. doi: 10.1097/MD.0000000000038766 (PMC11224875; doi:10.1097/MD.0000000000038766)
Supplement: Supplementary file 1 [file medi-103-e38766-s001.pdf]

# Supplementary File

## Global Contribution of Statistical Control Charts to Epidemiology Monitoring: A 23-Year Analysis with Optimized EWMA Real-life Application on COVID-19

**This file includes the:**

1. **Figure S1**
2. **Mathematical Structure of EWMA Control Chart**
3. **Detailed Discussion on Objetive 1**
4. **References**

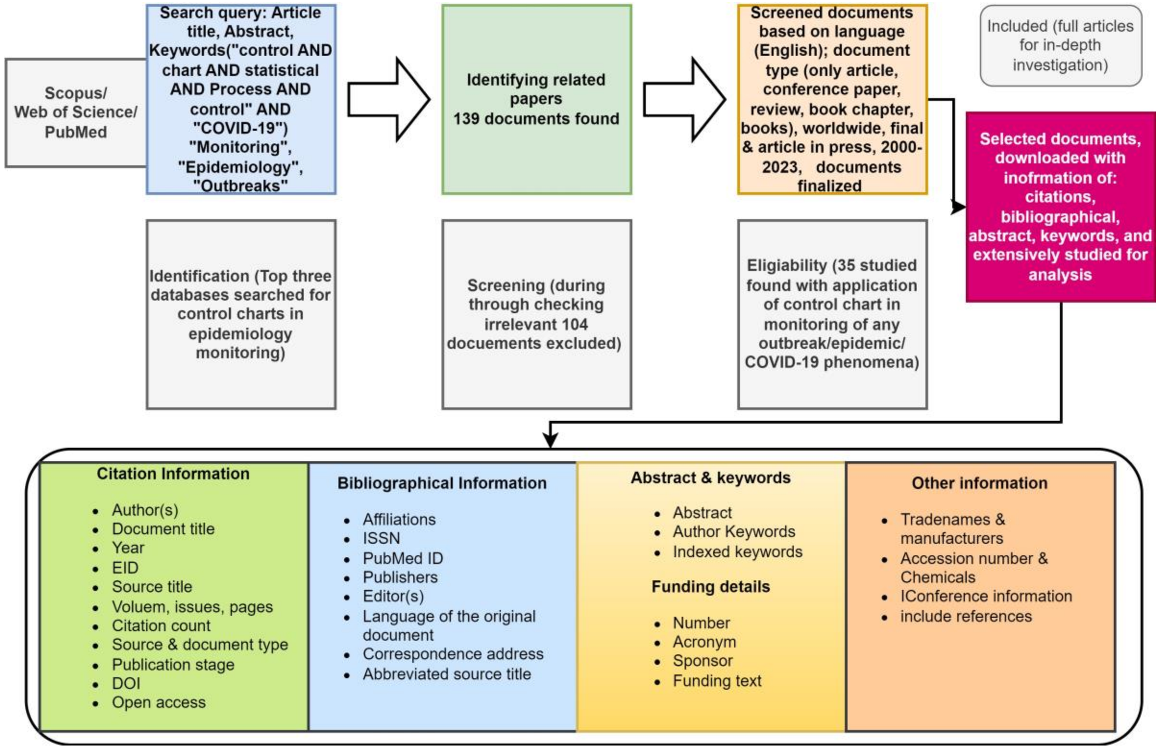

*Figure S1 Detailed description of the data collection process*

**Methods 2**

**Optimal EWMA Chart**

An EWMA chart is a statistical process control chart advocated by Roberts [1], a well-known scheme that is used to detect shifts in process parameters over time. Let a random variable $x_i$ represent the number of daily deaths, and $\lambda$ is a smoothing constant and satisfies the expression $0 < \lambda \leq 1$. Charting statistic ($Y_i$) is the current EWMA statistic, whereas, $Y_{i-1}$ is the previous EWMA statistic. Then $Y_i$ is defined as follows:

$$Y_i = \lambda x_i + (1 - \lambda) Y_{i-1} \text{ , where } i = 1,2,3, \ldots\ldots, n. \qquad (1)$$

The $Y_i$ is, therefore, a weighted average of all past and recent deaths, with more weight given to more recent observations. To calculate the control limits of the chart, we need to define the initial value $\mu_0$, which is the in-control process mean. We also need to calculate the standard deviation ($\sigma$) of the independent random observations. Finally, we must specify the width of the control limits, $L$, which is typically set at 3, but in our study during phase 1, $L = 2.87$ in order to attain $ARL_0 = 370$. The control limits of the EWMA chart are given as:

$$\left.\begin{aligned} UCL &= \mu_0 + L\sigma\sqrt{\lambda(2-\lambda)^{-1}[1-(1-\lambda)^{2i}]} \\ CL &= \mu_0 \\ LCL &= \mu_0 - L\sigma\sqrt{\lambda(2-\lambda)^{-1}[1-(1-\lambda)^{2i}]} \end{aligned}\right\}, \qquad (2)$$

If the EWMA statistic $Y_i$ falls outside these control limits, indicating a shift in the mean. The EWMA chart is handy for detecting small shifts in the mean, especially when $\lambda$ is small. If the number of deaths tends to infinity, then the term $[1-(1-\lambda)^{2i}]$ approaches to 1, in this case, the time-varying limits converted to asymptotic limits and are defined as follows:

$$\left.\begin{aligned} UCL &= \mu_0 + L\sigma\sqrt{\lambda(2-\lambda)^{-1}} \\ CL &= \mu_0 \\ LCL &= \mu_0 - L\sigma\sqrt{\lambda(2-\lambda)^{-1}} \end{aligned}\right\}, \qquad (3)$$

EWMA control chart applications in healthcare monitoring are widespread. The following studies can help understand the role of EWMA charts [2-4].

**Results and Discussion**

**More discussion on objective (1)**

***Charts application by category***

A wide range of approaches were found in the thorough examination of control chart applications in epidemiology, highlighting the various array of charts available for the surveillance of infectious illnesses. The word cloud in Figure 1, the name of all charts which are applied in different epidemiology monitoring phenomena. With 34% of applications, Shewhart Variable charts were the most popular option, closely followed by Shewhart attributes chart with 23%. With 19% of uses,

EWMA charts were quite important. Other versions, such as Hotelling's T-square and Cumulative Sum (CUSUM) charts, each contributed 11%. Even though they are still in their infancy, contemporary approaches like machine learning (ML) and deep learning (DL) charts only made up 2% of all chart applications see Figure 2(a). While in the context of tracking COVID-19, Shewhart Variable charts remained the most common, although their share was only 40%. Shewhart attribute charts application on second at 20%, with EWMA charts making up 16% of the total. When it came to COVID-19-specific applications, CUSUM and its variants (MEWMA & Hoteling's T-square) separately provided 4%, while ML and DL charts combined contributed 4%. This detailed analysis focuses on the particular chart preferences in the context of COVID-19 monitoring, offering insightful information on the changing patterns and approaches used to address the particular difficulties presented by the pandemic.

***Applications by year***

Control charts in epidemiology have a varied history. Between 2000-2010 it remains 28%, between 2010 and 2020 it shows 21%. Only in 2008, applications spiked to 9%, indicating a significant increase in recognition. Control chart applications have evolved, demonstrating their adaptability in various epidemiological scenarios. Focusing on COVID-19 data, 2021 and 2022 are crucial. Control chart applications rose to 18% in 2021, indicating greater attention on epidemic dynamics. Applications increased to 36% in 2022 and 41% in 2023, highlighting the urgent need to use control charts to monitor and analyze the worldwide health situation, see Figure 2(b). Control chart applications played a significant part in navigating infectious disease epidemiology throughout this transformative period during 23 years.

***Majority time variables monitored***

In epidemiological monitoring, many variables were investigated to monitor infectious diseases. In over 79% of studies other than COVID-19, counts of influenza, Salmonella, daily visits, infection rates, nosocomial infections, and patient numbers were significant variables. These different factors demonstrate control charts' applicability to a wide range of epidemiological scenarios and a comprehensive approach to understanding and treating infectious diseases beyond COVID-19. Where with 95% of variables contributing to COVID-19 pandemic monitoring and analysis, more attention was paid; to COVID infection rates, patient counts, symptoms, incubation period, positive cases, reported cases, mortality, and immunization rates were important variables, see Figure 3. COVID-19 investigations focus on a wider range of factors due to the pandemic's exceptional challenges, requiring a complex epidemiological surveillance strategy. The various variables employed in COVID-19 research highlight the global health epidemic and the necessity for specific tactics to understand and battle its intricacies.

***Study type***

Overall, 40% of control chart applications are longitudinal research. Retrospective studies account for 54% of applicants. A tiny but significant 6% of control chart uses come from mixed-method studies, showing their adaptability in epidemiology research. The distribution by study type of COVID-19 control chart applications shows significant trends. In 33% of COVID-19 uses, longitudinal studies are important. At 52%, retrospective studies dominate. Control charts can be adapted to varied study designs to capture the dynamic nature of the global health crisis, as shown by 10% of mixed-method studies.

## References


1. Roberts, S.W., *Control Chart Tests Based on Geometric Moving Averages.* Technometrics, 1959. **1**(3): p. 239-250.
2. Sparks, R., et al., *Understanding sources of variation in syndromic surveillance for early warning of natural or intentional disease outbreaks.* IIE Transactions, 2010. **42**(9): p. 613-631.

3. Mahmood, Y., et al., *Monitoring of three-phase variations in the mortality of COVID-19 pandemic using control charts: where does Pakistan stand?* Int J Qual Health Care, 2021. **33**(2).
4. Yupaporn, A. and S. Rapin, *EWMA control chart based on its first hitting time and coronavirus alert levels for monitoring symmetric COVID-19 cases.* 2021. **14**(8): p. 364-374.
5. Anwar, S.M., et al., *On mixed memory control charts based on auxiliary information for efficient process monitoring.* Quality and Reliability Engineering International, 2020. **36**(6): p. 1949-1968.
6. Jin, M., Y. li, and F. Tsung, *Chart allocation strategy for serial-parallel multistage manufacturing processes.* IIE Transactions, 2010. **42**: p. 577-588.
7. Yang, S.-F. and B.C. Arnold, *Monitoring Process Variance Using an ARL-unbiased EWMA-p Control Chart.* Quality and Reliability Engineering International, 2016. **32**(3): p. 1227-1235.
8. Zaman, B., et al., *An adaptive EWMA chart with CUSUM accumulate error-based shift estimator for efficient process dispersion monitoring.* Computers & Industrial Engineering, 2019. **135**: p. 236-253.
9. Areepong, Y. and W. Peerajit, *Integral equation solutions for the average run length for monitoring shifts in the mean of a generalized seasonal ARFIMAX(P, D, Q, r)s process running on a CUSUM control chart.* PLoS One, 2022. **17**(2): p. e0264283.
10. Rasheed, Z., et al., *Designing Efficient Dispersion Control Charts under Various Ranked Set Sampling Approaches.* Journal of Computational and Applied Mathematics, 2023: p. 115680.
11. Capizzi, G. and G. Masarotto, *An Adaptive Exponentially Weighted Moving Average Control Chart.* Technometrics, 2003. **45**: p. 199 - 207.
12. Anwar, S.M., et al., *An enhanced double homogeneously weighted moving average control chart to monitor process location with application in automobile field.* Quality and Reliability Engineering International, 2022. **38**(1): p. 174-194.
13. Anwar, S.M., et al., *A modified-mxEWMA location chart for the improved process monitoring using auxiliary information and its application in wood industry.* Quality Technology & Quantitative Management, 2020. **17**(5): p. 561-579.
14. Anwar, S.M., et al., *On mixed memory control charts based on auxiliary information for efficient process monitoring.* Quality and Reliability Engineering International, 2020.
15. Ou, Y., Z. Wu, and F. Tsung, *A comparison study of effectiveness and robustness of control charts for monitoring process mean.* International Journal of Production Economics, 2012. **135**: p. 479-490.
16. Rasheed, Z., et al., *An Efficient Robust Nonparametric Triple EWMA Wilcoxon Signed-Rank Control Chart for Process Location.* Mathematical Problems in Engineering, 2021. **2021**: p. 2570198.
17. Sanusi, R., N. Abbas, and M. Riaz, *On efficient CUSUM-type location control charts using auxiliary information.* Quality Technology and Quantitative Management, 2018. **15**: p. 87-105.
